# Robust OCC System Optimized for Low-Frame-Rate Receivers

**DOI:** 10.3390/s22165938

**Published:** 2022-08-09

**Authors:** Robert-Alexandru Dobre, Radu-Ovidiu Preda, Radu-Alexandru Badea

**Affiliations:** 1 Electronic Technology and Reliability Department, Politehnica University of Bucharest, 061071 Bucharest, Romania; 2 Telecommunications Department, Politehnica University of Bucharest, 061071 Bucharest, Romania

**Keywords:** visible light communication, optical camera communication, lighting, smartphone, video camera, LED

## Abstract

Light emitting diodes (LED) are becoming the dominant lighting elements due to their efficiency. Optical camera communications (OCC), the branch of visible light communications (VLC) that uses video cameras as receivers, is a suitable candidate in facilitating the development of new communication solutions for the broader public because video cameras are available on almost any smartphone nowadays. Unfortunately, most OCC systems that have been proposed until now require either expensive and specialized high-frame-rate cameras as receivers, which are unavailable on smartphones, or they rely on the rolling shutter effect, being sensitive to camera movement and pointing direction, they produce light flicker when low-frame-rate cameras are used, or they must discern between more than two light intensity values, affecting the robustness of the decoding process. This paper presents in detail the design of an OCC system that overcomes these limitations, being designed for receivers capturing 120 frames per second and being easily adaptable for any other frame rate. The system does not rely on the rolling shutter effect, thus making it insensitive to camera movement during frame acquisition and less demanding about camera resolution. It can work with reflected light, requiring neither a direct line of sight to the light source nor high resolution image sensors. The proposed communication is invariant to the moment when the transmitter and the receiver are started as the communication is self-synchronized, without any other exchange of information between the transmitter and the receiver, without producing light flicker, and requires only two levels of brightness to be detected (light on and light off). The proposed system overcomes the challenge of not producing light flicker even when it is adapted to work with very low-frame-rate receivers. This paper presents the statistical analysis of the communication performance and discusses its implementation in an indoor localization system.

## 1. Introduction

In recent years, optical wireless communication (OWC) has attracted numerous new research efforts, especially due to the limiting factors of present-time mainstream technologies, as well as to the overgrowing demand for mobile data communication and special purpose usage scenarios where increased implementation and deployment flexibility is required, which poses challenging constraints on already established solutions. One of the main driving factors behind OWC is the high annual growth rate of mobile traffic (up to 40% [1]), leading to the necessity of either bandwidth increases or spectral efficiency improvement. Because the spectrum efficiency is increasing at a much slower rate compared to the aforementioned traffic’s exponential growth, new high-frequency spectral bands are becoming an appealing alternative. With many desirable advantages, including available spectral bandwidth (from ca. 400 nm up to 1500 nm) coupled with a high energy efficiency [2,3,4], OWC is establishing itself more and more as a technology worthy of consideration. Recent advancements in both transmitter and receiver optical communication devices will make OWC deployments cost-effective, reliable, and secure. Therefore, OWC can be considered as a complementary solution to existing radio-based technologies for several classes of real-world utilization scenarios. With the spectrum utilization criterion in mind, we can identify several current OWC research directions: free-space optical (FSO) communications, visible light communications (VLC), optical camera communications (OCC), and light-fidelity (Li-Fi) [5].

FSO communications are based on a laser diode acting as transmitter and a photodiode as receiver, working on ultraviolet or visible light wavelengths, and offering a high-rate transmission over long distances. The drawback is the requirement of a strong correlation and position synchronization between the transmitter and receiver, making this solution relatively expensive. Atmospheric perturbation can also negatively impact the communication chain, but there are solutions to overcome these limitations [6,7,8,9,10].

VLC uses visible band signals generated by light emitting diodes (LED devices) as transmitters. The receiver is also photodiode-based. The proposed transmission distances are of medium range (meters to tens of meters), offering high data rates. Several indoor localization VLC-based proposals have been discussed [11,12,13,14,15]. One problem that emerges with navigation solutions based on VLC is the correlation between the LED field of view (FoV) coverage and receiver interference. LEDs with a larger FoV enable better receiver coverage, but at the same time, they increase the receptor signal interference. In this case, methods that evaluate signal-to-interference ratios [16] can be used to determine the real usable VLC coverage to eventually develop solutions for interference minimization.

OCC is a derivative technology from VLC that uses optical image sensors as signal receivers in the infrared or visible light bandwidths, being also referred as image-sensor communication [17,18]. OCC has many advantages compared to other OWC technologies. Since the receiver side consists basically of a ubiquitous (smartphone, surveillance, etc.) camera sensor, it is possible to quickly deploy larger-scale applications without requiring massive investments or logistical burdens to potential service users. OCC is one of the more promising OWC research fields, attracting much interest from applications related to the IoT, indoor navigation and positioning, intelligent transportation and control, etc. Nevertheless, in contrast to VLC, OCC can offer only modest data rates due to the limited sampling rate at the receiver, potentially camera focus loss, unstable frame rates, etc.

Increased interest in OCC has led to the publication of various surveys and research results about different aspects of the OCC data exchange chain. For example, [19] presents modulation techniques, advantages and limitations of OCC related to camera sampling rates, motion stabilization, and frame-rate variations. In [20], we can find a survey on OCC applications for intelligent transportation systems. Some of the more recent works on OCC include [21,22,23], where the authors reviewed OCC submissions to the IEEE 802.15.7m task group. A task group was formed in 2014 (TG7m) and is responsible for analyzing OCC transceivers, system architecture, and PHY and MAC layer specifications, having the IEEE.802.15.7m specification as an outcome. Updates on the standardization of OCC are available in [24]. Achievements related to TG7m can be found in [18].

The synchronization issues that are specific to OCC systems are thoroughly described in [23,25]. In [25], the authors highlight the difficulty of knowing the transmitted symbol timing directly in the receiver. This leads to a compromise between low-frame-rate compatibility and avoiding light flicker. To avoid flicker, the on–off switching of the light source must be accomplished fast enough, and to estimate the transmitted waveforms, a high sampling rate, and therefore a high frame rate camera, is required, leading to expensive systems and the limitation of broader public applications because these type of cameras are not available on smartphones. If low frame rates are used, the on and off switching of the light source must be slowed to allow the estimation of the transmitted waveform, leading to the occurrence of light flicker. One important advantage of the solution presented in [25] is that the system must discern between only two states of the light source, on or off, improving the communication robustness and reducing the difficulty of implementing the image processing algorithm. 

In [23], the authors review undersampled-based modulation schemes for OCC, highlighting the specific synchronization problems. The proposed solution is the use of a frame header. The main issue of this approach is that the image processing algorithm must now discern between three levels of brightness: light source on, light source off, and light source transmitting the frame header, thus reducing the noise margin, increasing the complexity of the image processing algorithm, and decreasing the robustness. Furthermore, to be able to detect the frame header, a slow shutter speed must be used. The shutter speed is the time interval in which the image sensor is exposed. Therefore, if slow shutter speeds are used (i.e., long exposure time intervals), it becomes very probable that the state of the transmitter LED will change during the exposure process, leading to uncertainty in detecting the LED state. In [26] a DPSK (differential phase shift keying) -based solution is proposed that uses another channel for error correction, thus requiring a direct line of sight (LoS) between the transmitter and the receiver and the identification of the light sources in the recorded frames, while still relying on three light states in the receiver for frame header detection. This paper solves the issues of the solutions proposed in [25,26], and the proposed solution can be adapted to any frame rate without producing light flicker, the receiver is relatively simple (it works only using two light states, light on and light off, offering the maximum robustness), and it does not require a transmitter–receiver LoS while still offering a fast enough system response for modern applications such indoor localization, which is demonstrated through experiments. The proposed system also uses fast shutter speeds.

Another category of OCC solutions is based on the rolling shutter effect. It relies on recording a light–dark pattern in a captured frame, and then the information is extracted from the pattern. These solutions for high robustness require direct capturing of the light source, the footprint of the light source on the image sensor to be large enough (needing to be in proximity of the light source, light sources with large dimensions, or the use of long focal lengths), and the resolution of the sensor to be high enough while being sensitive to motion during the acquisition process because it could lead to pattern distortion. The scanning direction of the sensor can vary from device to device; thus, the decoder must be designed so that the pattern is correctly decoded in any case. These requirements can be decreased by increasing the complexity of the image processing algorithms specific to these solutions. The solution presented in [27] is based on the rolling shutter effect, achieving a high bit rate but requiring a transmitter–receiver LoS, large image of the light source on the sensor, high-resolution image sensor, and relatively complex decoding algorithm. Our solution does not rely on the rolling shutter effect, and thus it does not have these disadvantages.

In summary, the main challenges that the system presented in this paper overcomes are:

- Working at low frame rates–the proposed system can use cameras capturing video at low frame rates (at most, 120 frames per second);
- Requiring auxiliary synchronization means between the transmitter and the receiver–the proposed communication is self-synchronized;
- Requiring a transmitter–receiver LoS–the proposed system can use reflected light and is invariant to the camera’s pointing direction;
- The occurrence of light flicker–the proposed communication code avoids the occurrence of noticeable light flicker phenomenon at any frame rate;
- Rolling shutter-determined challenges–the proposed system is not based on the rolling shutter effect;
- Requiring slow shutter speed to detect the frame header–the proposed system only uses two illumination states; therefore, it can use fast shutter speeds and avoid uncertainty in detecting the state of the transmitter LED; and
- Decreased robustness due to a low noise margin–the proposed system has to discern between only two light states (on or off) in the decoding process, thus increasing its robustness.

The main reason why this is important is because it represents a solution to making visible light communication available for mobile phones in a robust way. This paper extends the previously published results of [28,29,30,31,32,33,34,35,36]. Compared to the previously published papers, which mainly contained case studies on particular implementations of the system, this paper contains the generalized theoretical aspects of the proposed system (i.e., the detailed mathematical model of the communication and the mathematical expressions of the properties that the symbols require for obtaining the desired communication behavior) and the minimal duration symbols that meet the required properties, which were not previously presented or investigated, thus offering the highest possible bit rate of the system. In addition, this paper presents a structured and detailed way to implement the transmitter and the receiver, as well as a statistical analysis of the performance of the system if the receiver’s frame rate varies randomly, showing the robustness of the system and its implementation readiness in real-world applications owing to its short average decoding time. The main constraint of the system is the frame rate at which the receiver captures video content. Based on this, the design process is detailed further for a receiver that captures 120 frames per second. Then, derived versions of the system, designed for receivers capturing 60 frames per second and 30 frames per second, are briefly presented. In this way, one can design a similar system for a receiver capturing video at any frame rate.

Besides this introduction, this paper is structured as follows: Section 2 presents the proposed solution, offering mathematical models, explanations, examples, and illustrations for easing the understanding. Section 3 presents the results of the experiments run to demonstrate the invariance of the solution to the moments when the transmitter and receiver are started, thus demonstrating the solution to the specific OCC synchronization problem and the robustness of the proposed solution to the variation of the receiver’s frame rate. A laboratory test demonstrating the feasibility of the system in real-world conditions is also presented in this section. Section 4 discusses how the proposed solution can be used in an indoor localization system, describing one possible communication protocol. Two additional applications are briefly presented. The suitability of the proposed system to be used in an indoor localization solution is confirmed, owing to the fast system response time in the difficult bit-rate condition. Section 5 concludes the paper and highlights the future research opportunities.

## 2. Materials and Methods

The system is designed to work when the transmitter is represented by an LED ambient light system. In the past, ambient lighting solutions used mostly incandescent or fluorescent lights, both of which determine the occurrence of light-flickering at a frequency equal to twice the mains frequencies (i.e., 100 Hz or 120 Hz, depending on the region). At these frequencies, the light flicker is not noticeable to the human visual system, and it will stay unnoticeable if its frequency increases. Therefore, to maintain this desired feature, the transmitter part of the system should switch the light on and off at a frequency of at least 120 Hz. For ease of understanding, in this first part, it will be considered that the LED ambient lighting system, part of the VLC transmitter, is the only light source in the room. 

If an LED ambient lighting system in a room is switched on and off at a frequency of 120 Hz and a video camera records the lighting in that room at 120 frames per second, it means that the video camera will sample the light in the room at exactly the same state in every frame if the camera orientation does not change, therefore capturing the same brightness for every frame. In most real-life situations, small, natural movements of the camera caused by holding it in one’s hand will still lead to similar results because this does not determine a significant change in the average brightness of the captured frames. 

To allow the transfer of information, a change in the captured brightness from frame to frame must be made in a way that will not produce a perceptible light flicker. For this, the principles from binary phase shift keying (BPSK) modulation can be used. In BPSK, the logical 0 and logical 1 values in a binary message are represented by two separate phase states in the carrier signal, which is usually a sine wave. The two-phase values are typically 0° and 180°. When working with light, the notion of negative amplitude has no physical correspondent. Therefore, by adapting expressions of the waveforms of the two symbols that are characteristic to the BPSK modulation so that no negative values are obtained and adding the constraint that the light source should have only two possible states (on or off), the two symbols used by the proposed solution are mathematically described as follows:

(1) $$s_{k}\left(t\right)=\frac{1}{2}\left\{\mathrm{msign}\left[\sin\left(2\pi \cdot 120\cdot t-\frac{\pi }{2}+\bar{k}\pi \right)\right]+1\right\},\;k\in \left\{0,1\right\},\;t=\bar{0,T_{s}},$$

where T_s_ is the symbol duration, T_s_ = 1/120 s, and msign() is a modified version of the signum function:

(2) $$\mathrm{msign}\left(x\right)=\{\begin{array}{c} 1,\;x\geq 0\\ 0,\;x<0 \end{array}.$$

**Figure 1:**
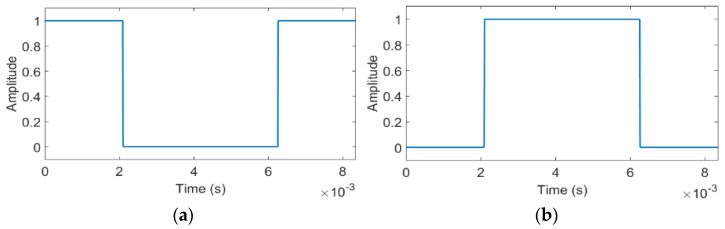
(**a**) The waveform for the $s_{0}$ symbol. (**b**) The waveform for the $s_{1}$ symbol.

The waveforms of the two symbols shown in Figure 1 are illustrations of the mathematical model. In the implementation of the system, they describe the state of the LED ambient lighting system, with 0 meaning it is switched off and 1 meaning it is switched on.

**Figure 2:**
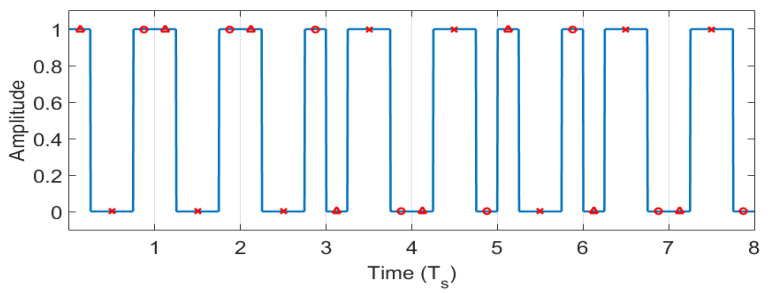
The waveform for the binary sequence 00011011 and the sampling points of interest.

A binary sequence can be transmitted by concatenating the waveforms of the corresponding symbols (e.g., $s_{0}$ for a bit equal to 0 and $s_{1}$ for a bit equal to 1). If the waveforms are sampled every T_s_ seconds, according to Equation (1), the resulting sequence has only two possible values: it is either equal to the transmitted sequence or equal to its negated value, no matter where the starting sampling point falls on the first symbol (i.e., no matter the moment when the receiver was started, as long as all the symbols are sampled). This property is illustrated in Figure 2, where all three possible cases of sampling are analyzed for the transmitted binary sequence equal to 00011011. If the sampling point is before the first quarter of the symbol’s waveform, the extracted sequence is 11100100 (the sampling points marked with triangles). If the sampling point is between the first quarter and the last quarter of the symbol’s waveform, the extracted sequence is 00011011 (the sampling points marked with crosses). If the sampling point is between the first quarter and the last quarter of the symbol’s waveform, the extracted sequence is 11100100 (the sampling points marked with circles). 

Therefore, in the first and last case, the negated value of the transmitted sequence was extracted, and in the second case, the extracted sequence is equal to the transmitted one. It should also be observed that the concatenation of the waveforms illustrated in Figure 1 could cause the occurrence of some other shorter pulses. Their duration is smaller than the largest pulse duration (1/240 s), and therefore they do not produce noticeable light flickering.

This property of extracting only the transmitted sequence or its negated value cannot assure a communication without an auxiliary synchronization method between the transmitter and the receiver, but it is valuable for the next stages in the design. Therefore, starting from the aforementioned symbols, a new set of two symbols must be designed, which can be uniquely identified no matter where the sampling moment is located during a symbol period to remove the requirement of the auxiliary synchronization. The waveforms for the new two symbols are obtained using the waveforms shown in Figure 1 by concatenating them according to a binary sequence and, in this way, conserving their property of not producing a noticeable light flicker.

Let the new two symbols be defined as two generic binary sequences of *i* bits:

(3) $$a=\left[a_{i-1},a_{i-2},\cdots ,a_{0}\right],a\in \left\{0,1\right\},\left\{i\in \mathbb{N}|i>1\right\},$$

(4) $$b=\left[b_{i-1},b_{i-2},\cdots ,b_{0}\right],b\in \left\{0,1\right\},\left\{i\in \mathbb{N}|i>1\right\}.$$

To remove the requirement of an auxiliary synchronization between the transmitter and the receiver, based on the property that the sampled sequence can be either the transmitted sequence or its negated value, the binary sequences that define the new two symbols must have the following properties:

(5) $$a,b\in {\left\{0,\;1\right\}}^{i},\;a\neq b,\;\bar{a}\neq b,$$

(6) $$\left[\left[a_{i-1-k},a_{i-2-k},\cdots ,a_{0}\right]⧺\left[a_{i-1},a_{i-2},\cdots ,a_{i-k}\right]\right]\cap^{\;}\left\{a,\bar{a},b,\bar{b}\right\}=\emptyset ,\;\forall k\in K,\;K=\left\{x\in \mathbb{N}|1\leq x\leq i-1\right\},$$

(7) $$\left[\left[a_{i-1-k},a_{i-2-k},\cdots ,a_{0}\right]⧺\left[b_{i-1},b_{i-2},\cdots ,b_{i-k}\right]\right]\cap^{\;}\left\{a,\bar{a},b,\bar{b}\right\}=\emptyset ,\;\forall k\in K,\;K=\left\{x\in \mathbb{N}|1\leq x\leq i-1\right\},$$

(8) $$\left[\left[b_{i-1-k},b_{i-2-k},\cdots ,a_{0}\right]⧺\left[a_{i-1},a_{i-2},\cdots ,a_{i-k}\right]\right]\cap^{\;}\left\{a,\bar{a},b,\bar{b}\right\}=\emptyset ,\;\forall k\in K,\;K=\left\{x\in \mathbb{N}|1\leq x\leq i-1\right\},$$

(9) $$\left[\left[b_{i-1-k},b_{i-2-k},\cdots ,a_{0}\right]⧺\left[b_{i-1},b_{i-2},\cdots ,b_{i-k}\right]\right]\cap^{\;}\left\{a,\bar{a},b,\bar{b}\right\}=\emptyset ,\;\forall k\in K,\;K=\left\{x\in \mathbb{N}|1\leq x\leq i-1\right\},$$

where {⧺} represents the concatenation operator. 

**Figure 3:**
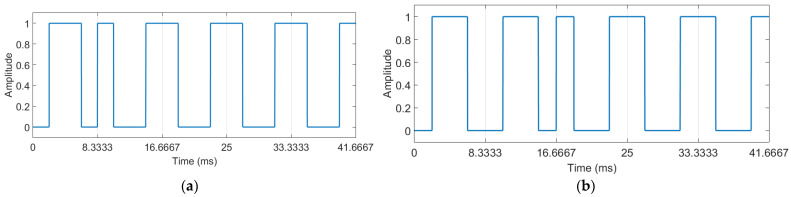
(**a**) The waveform for the symbol **a** (i.e., the binary sequence 10000). (**b**) The waveform for the symbol **b** (i.e., the binary sequence 11000).

The minimum value of *i* (i.e., the number of bits) for which such binary sequences exist is 5, numerically found by exhaustion search. In this case, there are 360 pairs of sequences that have the required properties. For example, one pair is given in Equation (10) and the corresponding waveforms are shown in Figure 3.

(10) a=[1,0,0,0,0], b=[1,1,0,0,0].

The detector requires five samples before a decision about what symbol was transmitted can be taken. Because of these properties, if the detector is not in proper temporal alignment with a symbol (i.e., from the five samples, some samples are from one symbol and the others from the next/previous symbol), the extracted binary sequence will not match any of the a, $\bar{a}$, b, or $\bar{b}$, sequences, and therefore no detection will take place. When the detector is in proper temporal alignment with a symbol (i.e., all the five samples are on the waveform of the same symbol), the extracted binary sequence will match one of the a, $\bar{a}$, b, or $\bar{b}$, sequences. Given the first property of the code, if the sampled sequence is either a or $\bar{a}$, the identified symbol is a. If the sampled sequence is either b or $\bar{b}$, the identified symbol is b. It is now demonstrated that by using binary sequences with the aforementioned properties, correct data transmission can take place without the need for any additional synchronization methods between the transmitter and the receiver, and thus the communication is self-synchronized. The transmitter and the receiver of such a system can be switched on independently, at any time moment. Correct transmission will take place starting from the symbol transmitted after the moment when both the transmitter and the receiver are on (i.e., if the transmitter is switched on after the receiver, correct transmission will take place starting from the first symbol; otherwise, naturally, the symbols that are transmitted while the receiver is off are lost). It can be observed in Figure 3 that if the symbols are sampled as shown in Figure 2, for each of them, the sampled value is either the corresponding binary sequence or its negated value. The duration of one symbol is 5/120 s, and therefore the bit rate is 24 bits per second.

**Figure 4:**
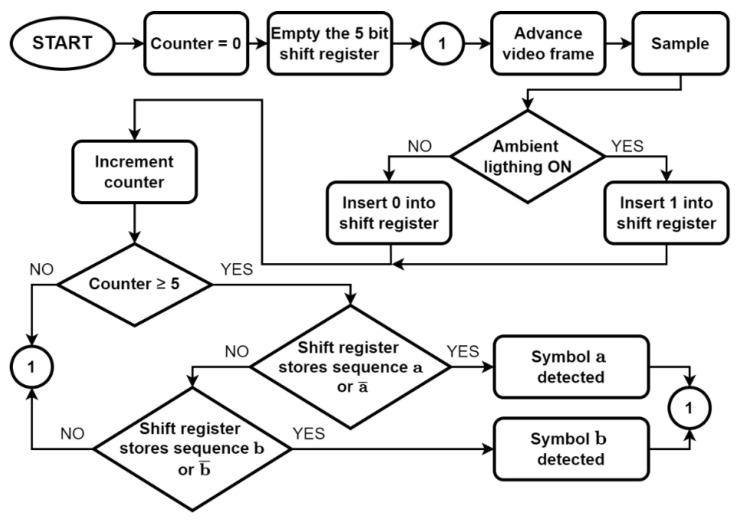
Flow diagram of the detection algorithm.

The flow diagram of the algorithm for the detection, implemented in the device containing the video camera (e.g., a smartphone), is presented in Figure 4. The receiver features a shift register with 5 bits. The system is initialized with an empty shift register and at least five samples must be acquired before the first detection attempt. The video frames are analyzed to determine if the light is on or off. For this description of the system, the light controlled by the VLC transmitter is considered the only source of light available. If the light is on in a frame, a bit equal to 1 is inserted in the shift register; otherwise, a bit equal to 0 is inserted. After five samples, the value stored in the shift register is compared with the reference sequences, a, $\bar{a}$, b, and $\bar{b}$, and the decision is made as explained above. Then, a new video frame is acquired and analyzed, the corresponding bit is inserted in the shift register, and the oldest entry in the register is removed. The new value is then compared again with a, $\bar{a}$, b, and $\bar{b}$, and the detection is completed as explained above.

The communication system described above can be designed for cameras capturing a lower number of frames per second. To maintain the property of not producing a noticeable light flicker, the waveforms for $s_{0}$ and $s_{1}$ cannot be simply correspondingly extended in time because the light pulses would become too long and the human visual system would perceive the light flickering. 

To design the system to work with cameras capturing 60 frames per second or 30 frames per second, it proceeds as follows. The waveforms corresponding to $s_{0}$ and $s_{1}$ that conserve all the presented properties, but are designed for a decoder capturing 60 frames per second, are denoted further with $s_{0,60}$ and $s_{1,60}$, and are obtained as follows:

(11) $$s_{0,60}=s_{0}⧺s_{0},$$

(12) $$s_{1,60}=s_{1}⧺s_{1},$$

where $s_{0}$ and $s_{1}$ are as illustrated in Figure 1. Because the duration of the symbol that encodes one bit is doubled, the consequence is a reduction of the bit rate to 12 bits per second. In a similar manner, the waveforms, for 30 frames per second, $s_{0,30}$ and $s_{1,30}$, are obtained with:

(13) $$s_{0,30}=s_{0,60}⧺s_{0,60}=s_{0}⧺s_{0}⧺s_{0}⧺s_{0},$$

(14) $$s_{1,30}=s_{1,60}⧺s_{1,60}=s_{1}⧺s_{1}⧺s_{1}⧺s_{1},$$

with the consequence of obtaining a bit rate equal to 6 bits per second.

In countries where the main frequency is 50 Hz, the classic ambient lighting systems flicker at 100 Hz, and this is still unnoticeable to the human visual system. Therefore, to design the communication system for video cameras capturing 100, 50, or 25 frames per second, the process is similar and needs only the modification of the $s_{0}$ and $s_{1}$ waveforms, dilating them in time to have a new duration of 1/100 s. This is achieved by changing the frequency of the sine wave in Equation (1) from 120 Hz to 100 Hz and the T_s_ value from 1/120 s to 1/100 s.

## 3. Results

### 3.1. Testing the Proposed System for Invariance to the Moment When the Receiver Is Started

In general purpose OCC applications, the preferred receivers should be represented by smartphones. In this way, all the required hardware is already available and only a software for the receiver needs to be developed. A practical VLC system must allow the user to receive the transmitted information, no matter when the user chooses to start the receiving software application. Therefore, a practical VLC system should be invariant to the moment when the receiver is turned on.

Numerical analysis was performed to determine the invariance of the proposed communication system to the starting moments of the receiver and the transmitter. All the 360 pairs of symbols on 5 bits that have the required properties (i.e., described in Equations (5)–(9)), were tested in the following way: a 500 bits binary pseudorandom sequence was generated, then each bit of it was encoded with symbol **a** or symbol **b** (e.g., 1→**a**, 0→**b**), where **a** and **b** are 5 bits binary sequences from the test set. After this, the waveform for the whole transmission was generated starting from the binary sequence that had so far resulted using the following correspondence: the bits equal to 0 were represented using the waveform for $s_{0}$ and the others were represented using the waveform for $s_{1}$. The moment when the receiver was switched on was randomly chosen, then the waveform was sampled with a sampling frequency of 120 Hz, modelling a 120 frames per second video acquisition. The experiment was repeated 100 times for every pair (i.e., 100 random start moments). The numerical results showed that the transmission was correctly decoded starting from the next full symbol after the moment where the receiver was started (i.e., only the symbol whose transmission already started when the receiver was switched on was lost and, naturally, all the symbols before it). This demonstrates that the system is self-synchronizing with the data transmission and shows its ability to identify the symbols correctly and automatically, no matter when the receiver is started. The experiments were repeated in the corresponding way when the system was designed for cameras capturing 60 frames per second and again for 30 frames per second, using the corresponding symbols, $s_{0,60}$ and $s_{1,60}$ for 60 frames per second and $s_{0,30}$ and $s_{1,30}$ for 30 frames per second, with the same conclusion: the system is invariant to the moment when the receiver is started.

### 3.2. Testing the Proposed System’s Resistance to the Variation of A Video Camera’s Frame Rate

In this subsection, we study the behavior of the decoder if the video recording at the decoder side is affected by frame rate variation errors. The expected behavior of the decoder is to be able to extract the transmitted binary code correctly and in a reasonable amount of time.

The transmitted signal *S* is obtained by transmitting an 18 bits binary sequence *c* an *n* number of times in a loop. For frame synchronization purposes, *c* is composed of six preamble bits “111110”, followed by three digits encoded using binary-coded decimal (12 bits), being able to transmit any decimal number between 0 and 999. Every bit of *c* is encoded using one of the 360 pairs of symbols **a** and **b**, one of the pairs being presented as example in Figure 3. All the other pairs can be found by respecting the constraints in Equations (5)–(9). The waveforms for **a** and **b** have each a length of 10,000 samples in simulation to allow a proper sampling resolution and the modeling of the frame rate variation.

The proposed maximum acceptable decoding time of 15 s has been obtained as follows: the duration of a symbol (i.e., **a** or **b**) is $t_{\mathrm{symbol}}=\frac{i}{f_{0}},$ where *i* is the size of the symbol in bits (i.e., *i* = 5 for the proposed code) and *f*_0_ is the nominal frame rate. One transmission of the room identification code (18 bits, including preamble) is $t_{\mathrm{RIC}}=18\cdot t_{\mathrm{symbol}}.$ To transmit it *n* = 20 times, it will require a time interval of: $t=n\cdot t_{\mathrm{RIC}}=18\cdot n\frac{i}{f_{0}}$. For a frame rate of *f*_0_ = 120 frames per second, the obtained value for *t* is *t* = 15 s.

We simulate the video recording of the light intensity using a smartphone camera that introduces frame rate errors around a nominal frame rate $f_{0}$. To achieve this, the signal *S* is sampled using a sampling rate of $f_{S}=f_{0}(1-e)$, where *e* is the frame rate error. We will evaluate the most likely scenario to occur in practice, where the frame rate is affected by a variable error rate *e*, resulting in a variable frame rate, but is always lower than $f_{0}$ (i.e., the camera is “lagging”, and *e* is always positive). The sampling starts at a random moment inside the time window corresponding to the first transmission of symbol *c*.

The decoding is completed using a sliding window approach by searching for the reference sequences a, $\bar{a}$, b, and $\bar{b}$, in the sampled signal. If either a or $\bar{a}$ is found, the decoded bit will be “0”. If b or $\bar{b}$ is found, the decoded bit will be “1”. The resulting decoded bits are stored in a binary vector **d**. Next, we search for the preamble code “111110” in vector **d**. The next 12 bits after the preamble will be compared to the corresponding bits that have been transmitted. If they match, we consider that a correct detection has been completed and we increment the counter for correct detections.

To measure the detection performance of our system, the following parameters are evaluated for *n* = 20 repetitions of symbol *c* for every pair of symbols **a** and **b**:

- Average time until the first correct detection of symbol *c* is performed by the decoder (ADT);
- Maximum time until the first correct detection of *c* (MaxDT);
- Average number of correct detections of *c* (AD); and
- Minimum number of correct detections of *c* (MinD).

We have considered maximum frame rate error values $e_{\max}$ in the range 0.1% to 2%, with a step size of 0.1%. For every given maximum error $e_{\max}$, the frame rate error *e* takes random values in the interval $\left[e_{max}-0.1;e_{max}\right]\%$.

The test is repeated for every pair of symbols **a** and **b**, with 1000 different random values for the number transmitted (from 0 to 999), 100 different start moments (within the time interval corresponding to the first transmission of *c*), and the resulting symbol *c* is transmitted *n* = 20 times in a loop to achieve statistical relevance. We have chosen *n* = 20 because this would correspond to a maximum decoding time of 15 s for a nominal frame rate of 120 frames per second. Any longer decoding time could be considered unreasonably long. For every test, the time until the first correct detection of *c* and the number of correct detections out of *n* = 20 is experimentally determined, and the ADT, MaxDT, AD, and MinD parameters are evaluated for every pair of symbols.

**Figure 5:**
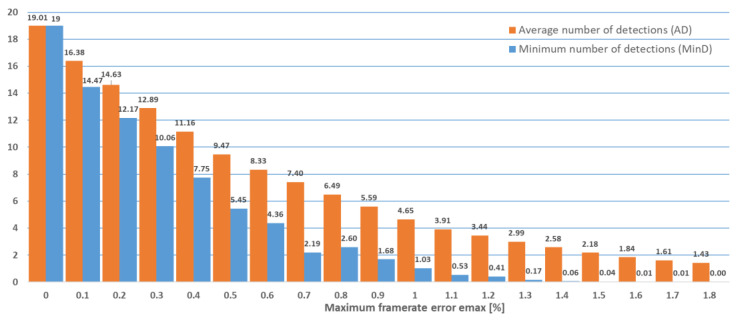
Minimum number of detections (MinD) and average number of detections (AD) out of a maximum of 20 (120 frames per second case).

Figure 5 shows the average values (calculated over all 360 pairs of symbols) of the AD and MinD parameters out of *n* = 20 maximum possible detections for every pair. The figure shows that both the AD and MinD values decrease with higher maximum error rates. We can also see that the average MinD is higher than 1 for maximum error rates lower than 1%. There are some codes that can be used for even higher error rates, up to 1.8%, as can also be seen in Table 1.

To better show the performances of the different codes regarding ADT and MaxDT, Table 1 presents the statistical analysis of all the 360 different variants of the code pairs (**a** and **b**) used to encode the binary values “0” and “1”. For $e_{\max}>1.8\%$, no transmission was correctly decoded.

Table 1 shows that the ADT has acceptable values lower than 3.45 s and even as fast as 0.97 s. The MaxDT has values under 5 s for maximum frame rate errors of, at most, 0.6%, and in very harsh conditions, it is under 15 s.

**Figure 6:**
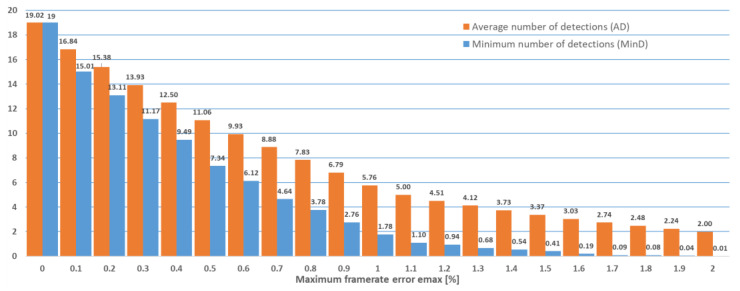
Minimum number of detections (MinD) and average number of detections (AD) out of a maximum of 20 (60 frames per second case).

**Figure 7:**
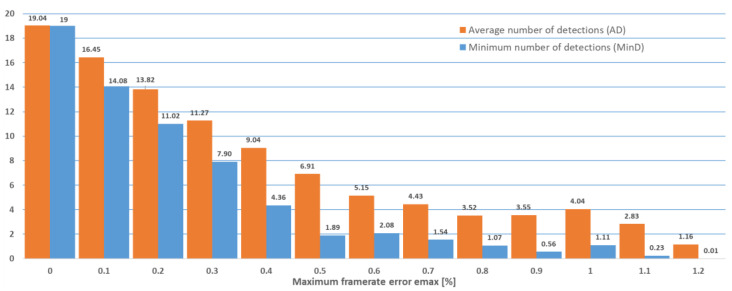
Minimum number of detections (MinD) and average number of detections (AD) out of a maximum of 20 (30 frames per second case).

The experiments described above were repeated for the case when cameras capturing 60 frames per second were used, as summarized in Figure 6 and Table 2. The case when cameras capturing 30 frames per second were used is summarized in Figure 7 and Table 3.

It can be observed that not all the 20 transmitted *c* sequences are decoded correctly even if the error rate of the receiver is set to 0%. This happens because in the experiments, the transmitter and the receiver are not synchronously started. The receiver is started at a random moment during the transmission of the first *c* sequence, and therefore a significant part of it could be lost, which explains why the number of correctly decoded sequences is below 20.

### 3.3. Laboratory Tests

**Figure 8:**
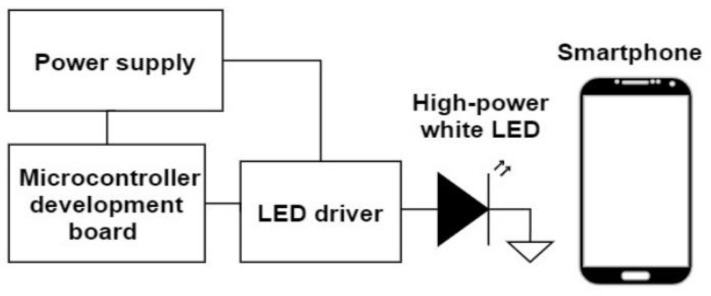
The block diagram of the laboratory prototype used for testing the communication system.

A prototype of the system was implemented to demonstrate its feasibility in real-world conditions. A white LED was used as the ambient light source, also playing the role of the transmitter. A video recording of the reflected light on a wall was captured using a handheld Samsung Galaxy S10 smartphone recording 30 frames per second at a resolution of 720 × 1280 pixels. The block diagram of the laboratory prototype is shown in Figure 8. 

**Figure 9:**
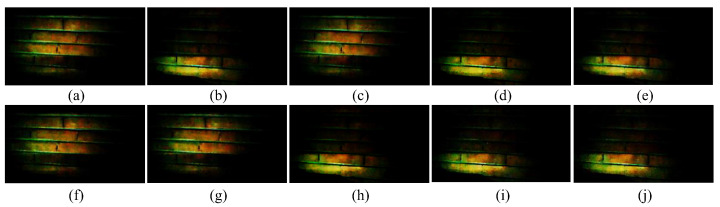
Ten consecutive video frames recorded during the evaluation of the prototype. For the ease of understanding, the chronological order of the frames was marked with letters from (**a**–**j**).

Ten consecutive video frames from the captured video are illustrated in Figure 9 to offer the reader details about the functioning of the system. The number of images that are presented is chosen to be relevant to the reader while avoiding overcrowding the paper. With the presented images, the detection of two consecutive symbols can be explained. The rest of the symbols contained in the whole video sequence are decoded in a similar way.

The a sequence (corresponding to the “1” symbol) used in the experiment was 10100 and the b sequence (corresponding to the “0” symbol) was 11000. The steps of the image processing algorithm to detect the transmitted code are as follows:

- For every video frame *i,* a row of pixels is extracted. For the prototype, it was chosen as the row number 240;
- This row of pixels is converted from the RGB to the YCbCr color space and only the luminance (Y) component is further processed;
- The mean luminance *M_i_* of the row of pixels of the current frame *i* is computed. Choosing a row rather than the entire frame is preferred because each row of a frame is exposed separately (at different moments in time), as can be observed in Figure 9. The upper side of the frames and the lower side of the frames have opposite brightness levels. This is because the LED’s state changed during the process of capturing each frame;
- A threshold *T* is calculated as the average over every frame of the means of the same central row of pixels. For the experiment, the threshold was equal to 75;
- *M_i_* for the current frame is compared to the threshold *T.* If it is greater than *T*, we decide the that the LED is on (i.e., the decoded bit value is “1”). Otherwise, we decide the LED is off (i.e., the decoded bit value is “0”);
- The above steps are repeated for every frame, obtaining a sequence of bits equal to the number of frames of the video; and
- The obtained sequence of bits is further decoded as described in Section 2.

The mean luminance values obtained for the frames shown in Figure 9, in order, are equal to: 102.2, 51.8, 108.9, 49.4, 57.7, 117.3, 111.1, 51.6, 56.2, and 53.2, and they identify the LED states as on, off, on, off, off, on, on, off, off, and off, extracting the code 1010011000. By applying the decoding algorithm, as presented in Section 2, on this extracted binary sequence, the **a** sequence can be identified (the first 5 bits), followed by the **b** sequence. Therefore, with the ten presented frames, the transmission of the two bits binary sequence 10 can be identified. This is a very simple image processing algorithm that still proves the feasibility of the system in real-world conditions. As stated, the optimal image processing algorithm for determining the two possible light states, on or off, can be further developed and is open for further research.

## 4. Discussion

The communication system proposed in this paper is remarkable because it sets a base for making OCC available to the broader public, allowing the development of general-purpose applications with this technology by facilitating the use of smartphones as receivers. Currently, OCC solutions revolve around using very high frame rate video cameras, which are very expensive and not integrated in a system designed for facile software development, or they are based on the rolling shutter effect, which makes the system very sensitive to camera motion and usually requires pointing the camera to the light source, which must have large dimensions on the captured frame for robustness, while the recording device needs to offer high resolution images to allow for proper decoding of the light pattern. 

To be able to receive a one-shot transmission, the receiver must be started before or at limit in the same moment as the receiver so that no symbols are lost due to the receiver still being off while the transmission has already begun. This situation should be avoided when developing applications that use the proposed system (or any other simplex communication system). The solution to this problem is that the transmitter should send information repeatedly and the transmitted information should be sent in a way that the start of the message can be uniquely identified (e.g., by using a preamble). Therefore, an even higher-level protocol of communication should be used to feature this property, too.

The main limitation of the proposed system is the bit rate, 24 bits per second for 120 (or 100) frames per second, and it scales linearly down with the frame rate of the camera that is used in the receiver. The system is not designed for exceeding the bit rate of other OCC systems, but rather, to be robust, easily implemented with smartphones as receivers, and easily integrated to be an open platform to help developers to build OCC applications for the broader public.

The system was presented considering that the ambient LED light that transmits the information is the only light source available. In this case, when this light is off, there is no light in the room. Depending on the application, daylight can be present, which is a continuous light. This is a challenge for all the OCC systems. Therefore, the image processing algorithm must be able to distinguish between daylight and ambient light either by intensity or by spectral characteristics. Image processing algorithms that can make the detection more robust in these lighting situations represent another research topic that can contribute to OCC applications.

One of the applications that can be elegantly implemented using the proposed system is indoor localization and navigation. In this application, all the ambient lights from each room in a building will transmit a unique code which identifies the room (e.g., the room number) repeatedly, and it marks the start of the message with a preamble. This application does not need a high bit rate because the messages are short (the preamble and the room identification code (RIC)), and, naturally, it allows the cyclical sending of the same message by all the VLC transmitters in a room (i.e., the ambient lighting). One way to encode the room number *xyz* is the following:

(15) $$s_{xyz}=\left[111110⧺x_{\mathrm{BCD}}⧺y_{\mathrm{BCD}}⧺z_{\mathrm{BCD}}\right],$$

where $x_{\mathrm{BCD}}$, $y_{\mathrm{BCD}}$, and $z_{\mathrm{BCD}}$ are binary-coded decimal (BCD) representations of the values of *x*, *y*, and *z*, where the room number is written as:

(16) xyz=100·x+10·y+z.

For example, the room number 319 is encoded as 111110001100011001, where 111110 is the preamble, 0011 is the BCD representation of 3, 0001 is the BCD representation of 1, and 1001 is the BCD representation of 9. In this way, 1000 rooms can be encoded. There are 18 bits in a message; therefore, using a receiver capturing video at 120 frames per second, the detection is completed in less than 2 s in the worst-case scenario of temporal alignment with the message (i.e., the receiver is started when the second bit from the preamble is sent), as discussed in the previous section of the paper. The largest buildings of the world have approximately 1000 rooms and could be fit with this system. For example, the Palace of the Parliament in Romania has 1100 rooms.

Using the camera fitted on a smartphone and a software application, the detector can sense the variations of light in a room and extract the room number, then show the user their current location on a map of the building. It can also help the user to get to any room in the building by monitoring their current position in the described way and providing instructions for navigation. A room level localization resolution is enough for general purpose indoor localization and navigation. 

A similar application is in the case of museums. To offer visitors more information about the exhibits and increase engagement, the lighting system that illuminates each exhibit can transmit an exhibit identification code, similar to the room code discussed above. The visitors can then use their smartphones to detect the code of an exhibit and automatically be sent to a page where more information or other interactive content is shown. The experience is similar to scanning a QR code, but the light is scanned instead. This is better than using QR codes because light is a natural presence in the exhibits of a museum, while the presence of QR codes is artificial.

Another application that could be implemented with the proposed system is video copyright protection. The transmitter system can be implemented in lighting gear or monitors mounted on the stage during a concert to transmit a code. If someone in the public tries to record a video of the performance, the code can be detected by the recording device, and then the device can learn that the recording is restricted and stop recording automatically.

When developing applications with the proposed system, the shutter speed of the video camera should be set to at least 1/8000 s [27] to avoid averaging the brightness for too long a time, which leads to uncertainty about the state of the LED lighting system. Nowadays, smartphones can record video with shutter speeds of 1/24,000 s (e.g., Samsung Galaxy S10–released in February 2019); therefore, fast shutter speeds are readily and commonly available, unlike very high frame rates. The advantage of the proposed method over others that use fast shutter speeds is that it does not rely on the rolling shutter effect, and therefore it removes the communication’s sensitivity to camera movement and does not necessitate a direct view of the light source.

Finally, a comparison of the proposed solution with other OCC solutions is provided in Table 4.

## 5. Conclusions

The paper presents an OCC system with properties that facilitate the development of robust applications using readily available devices (i.e., smartphones capturing video at low frame rates). The proposed solution is self-synchronized, invariant to the moment when the transmitter and receiver are started and to the camera pointing position, and it can function with reflected light, and thus it does not require a line of sight towards the light source because it does not need its detection and identification in the decoding algorithm. The solution is not based on the rolling shutter effect, removing the high sensitivity to camera movement that exists in such systems, as well as the requirement of high-resolution sensors. These properties represent additional important benefits. It requires the identification of only two states of the lights source, maximizing the robustness. All the above characteristics and the acceptable system response time demonstrated through experiments make the solution a base for developing general-purpose OCC applications for the broader public.

The mathematical model of the system and of the properties that must be met by the symbols used for communication are given and explained, along with the modifications that should be made to adapt it to various receivers’ frame rates. Illustrations and examples are also given at each step. 

The system was modeled and thoroughly tested for invariance to the moment when the transmitter and the receiver are started and for resistance to the variation of the recorder’s frame rate. The results show that the system is invariant to the moment when the receiver and transmitter are started for all the code pairs meeting the required and presented properties, as it is self-synchronizing. The system presents good resistance to frame rate variation, with the results being presented for 120, 60, and 30 frames per second.

It is common for OCC systems to feature a relatively low bit rate. The presented system has a bit rate of 24 bits per second for a 120 frames per second receiver, scaling down linearly with the frame rate. The bit rate is sufficient to assure short average detection times for modern applications, and the system excels by removing the common constraints of OCC systems described above and by its ease of implementation. Further, we have also presented a proposal for a higher-level communication protocol that can be used when addressing indoor localization and navigation services to large and very large buildings. Two other applications were also discussed. 

The system also features an open processing block—the image processing algorithm responsible for evaluating the frames’ brightness—that can represent a potential subject for future research.

## Figures and Tables

**Table 1:** Statistical analysis of the ADT and MaxDT (120 frames per second case).

*e*_max_	Percentage of Code Pairs That Can Be Used for Successful Decoding	Average ADT (Seconds)	Average MaxDT (Seconds)
0.5%	100%	0.97	2.96
0.6%	100%	1.18	4.49
0.7%	84.72%	1.35	7.39
0.8%	98.61%	1.61	6.98
0.9%	83.89%	1.78	8.25
1%	70.83%	2.13	9.96
1.1%	45.28%	2.38	10.74
1.2%	39.44%	2.58	11.32
1.3%	16.39%	2.65	11.51
1.4%	5.83%	2.88	11.96
1.5%	3.98%	2.95	12.09
1.6%	0.83%	2.74	11.03
1.7%	0.56%	3.03	11.58
1.8%	0.28%	3.45	13.42

**Table 2:** Statistical analysis of the ADT and MaxDT (60 frames per second case).

*e*_max_	Percentage of Code Pairs That Can Be Used for Successful Decoding	Average ADT (Seconds)	Average MaxDT (Seconds)
0.5%	100%	1.97	5.72
0.6%	100%	2.22	6.8
0.7%	100%	2.5	8.32
0.8%	99.72%	2.96	11.6
0.9%	96.94%	3.51	13.91
1%	84.72%	3.97	17.38
1.1%	70%	4.31	18.93
1.2%	66.38%	4.67	20.28
1.3%	56.11%	4.94	21.13
1.4%	48.33%	5.31	22.66
1.5%	36.94%	5.66	23.03
1.6%	18.61%	5.82	23.55
1.7%	9.16%	6.2	23.16
1.8%	7.5%	6.57	23.91
1.9%	4.16%	6.59	24.52
2%	0.83%	7.38	25.81

**Table 3:** Statistical analysis of the ADT and MaxDT (30 frames per second case).

*e*_max_	Percentage of Code Pairs That Can Be Used for Successful Decoding	Average ADT (Seconds)	Average MaxDT (Seconds)
0.4%	100%	5.47	17.95
0.5%	88.88%	9.14	37.46
0.6%	84.16%	8.41	31.62
0.7%	78.33%	9.09	34.55
0.8%	78.33%	12.04	40.06
0.9%	45.55%	10.70	41.21
1%	70.55%	14.54	44.18
1.1%	21.38%	12.46	46.28
1.2%	0.83%	15.09	52.11

**Table 4:** Comparison between the proposed solution and other available OCC solutions.

Solution	Rolling Shutter Drawbacks	Requires A Source–Receiver LoS	Requires Extra Synchronization Channel	Light States Used in the Decoder	Flicker Occurrence Possibility
[23]	No	Yes	No	3	No
[25]	No	Yes	No	2	Yes
[26]	No	Yes	Yes	3	No
[27]	Yes	Yes	No	2	No
Ours	No	No	No	2	No
